# Gross and Histopathology of COVID-19 With First Histology Report of Olfactory Bulb Changes

**DOI:** 10.7759/cureus.11912

**Published:** 2020-12-04

**Authors:** George S Stoyanov, Lilyana Petkova, Deyan L Dzhenkov, Nikolay R Sapundzhiev, Iliyan Todorov

**Affiliations:** 1 General and Clinical Pathology/Forensic Medicine and Deontology, Medical University of Varna, Varna, BGR; 2 Otolaryngology, Medical University of Varna, Varna, BGR; 3 Infectious Diseases, Parasitology and Dermatovenerology, Medical University of Varna, Varna, BGR

**Keywords:** covid-19, anosmia, pneumonia, pathology, autopsy, olfactory bulbitis

## Abstract

In nearly a year since the first reported cases of coronavirus disease 2019 (COVID-19), caused by the severe acute respiratory syndrome coronavirus 2 (SARS-CoV-2), a lot has been established about the virus. Correlates in regards to the biology and cellular effects of SARS-CoV-2 have brought a lot of explanations to the clinical manifestations of the disease and possible therapeutic modalities. However, despite the discoveries made, the tropism of SARS-CoV-2 has not yet been fully established, nor have all the clinical aspects of COVID-19. Herein we report the gross and histological findings in two diseased patients. Apart from the already established pulmonary and vascular changes caused by SARS-CoV-2, we report the presence of histological changes of the olfactory bulbs and frontal lobes of the brain, which may present as a correlate for COVID-19 related anosmia. The olfactory bulbs histologically showed necrotizing olfactory bulbitis. As both the olfactory bulb and frontal lobe of the cerebrum are key areas of olfaction, we believe that this tropism of SARS-CoV-2 may be key to the development of anosmia and not changes within the nasal cavity.

## Introduction

In nearly a year since coronavirus disease 2019 (COVID-19) was first identified, multiple aspects of the disease have been established [1,2]. However, the morphological bases have so far been poorly studied, with most research focusing on the pulmonary and vascular effects of the virus [3-5].

One of the most well clinically established, but to our knowledge unreported, morphological aspects of the virus is the development of hypo and anosmia as well as hypo and ageusia [6-8].

Herein we present two autopsy case reports of a Caucasian male in his 70s and a Caucasian female in her 40s, which to our knowledge are the first histopathological reports of changes in the olfactory bulb.

## Case presentation

The first patient presented to our institution with a three-day history of shortness of breath, dry cough, loss of appetite, muscle pain, and fever of up to 39^o^C. Previous medical history included myasthenia gravis-like symptoms for which the patient was treated with pyridostigmine bromide and cerebral cortical atrophy of vascular genesis. At the time of presentation, the patient was quarantined in the same household with a COVID-19 positive person, but he had opted out of being tested.

On examination, the patient had signs of severe illness with severe dyspnea, facial cyanosis, and saturation of oxygen (SpO2) 78%. The patient was immediately transferred to the intensive care unit, however expired shortly after admission.

The second patient presented to our institution for non-COVID-19 related symptoms as she had multiple comorbidities, including previous medical history for malignancy. During her hospital stay, she developed shortness of breath, a fever of up to 38^o^C, and a dry cough. Three days after her test came back positive the patient developed severe hypoxia and expired.

As the cases were deemed high-risk ones a full safety protocol was initiated with a three-member team performing the autopsy - one hall attendant and two pathologists. Full protective gear was used as well as N-95 facial masks and protective helmets. The autopsies were video documented in full, from the point of view of the dissecting pathologist.

Gross findings

On examination, before the autopsy, only facial cyanosis was noticed in both patients. Section of the thoracic cavity revealed the lungs were contracted towards the hilus, cyanotic, and with severely increased weight (case one: left lung 1000 grams, right lung 1100 grams; case two: left lung 980 grams, right lung 1000 grams ). In both patients, the lungs fully sunk when submerged underwater (positive lung float test). On cross-section, the lungs were diffusely consolidated, without any thrombi visible in the vasculature (Video 1). The tracheas and bronchial trees were edematous with severe erythema in the interchondral areas of the mucosa.

**Video 1:** Sectioning of the right lung. Diffuse consolidation of the parenchyma. Video is a trimmed-out part of the field of view of the whore recording.

Samples for histology were collected from all internal organs. Despite anosmia not being mentioned as one of the presenting symptoms of the patients, the olfactory bulbs were also collected for histological evaluation. Collected specimens for histology were processed in a standard manner, embedded in paraffin, and stained with hematoxylin and eosin.

Pulmonary histopathology

**Figure 1:**
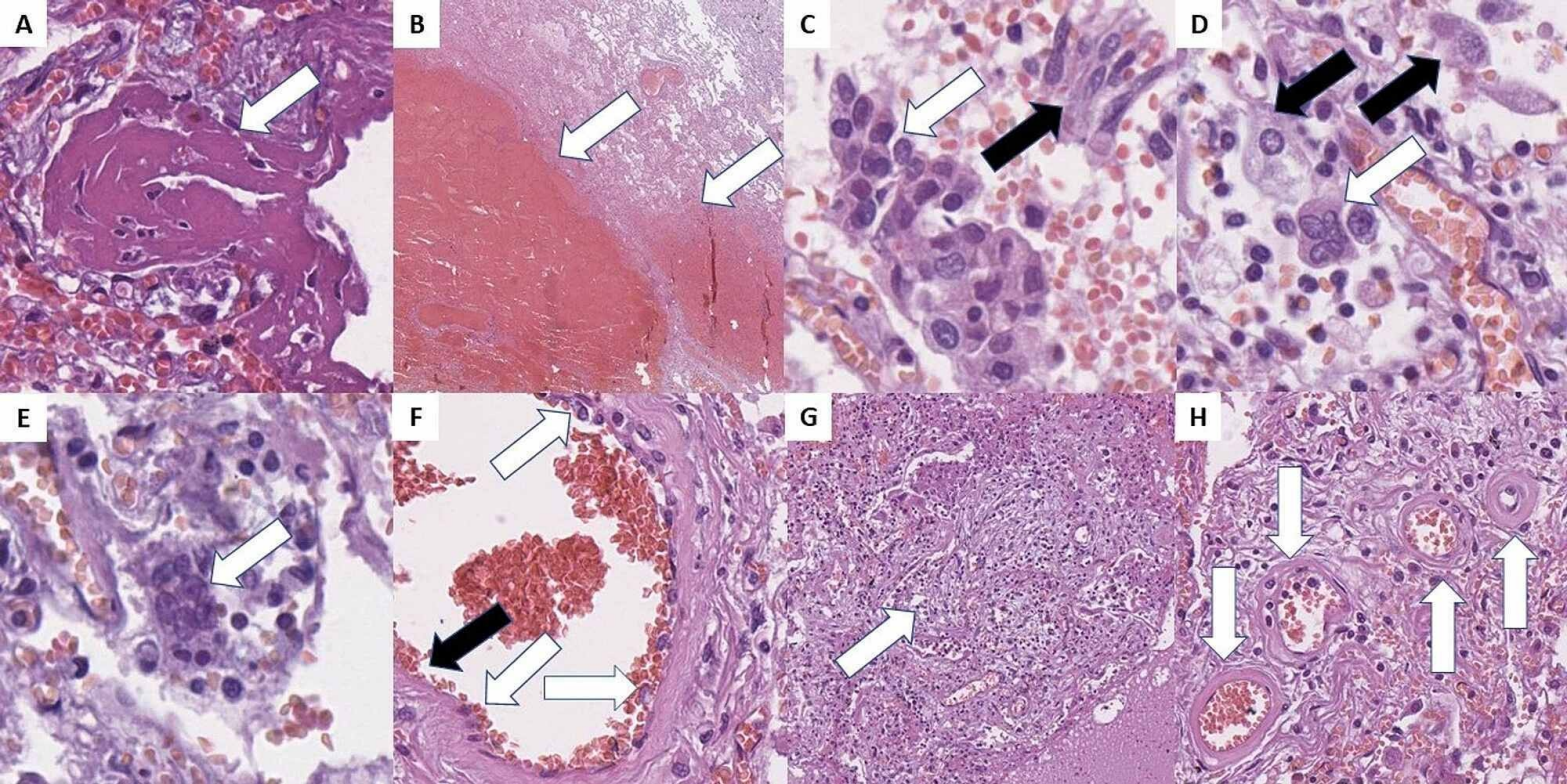
COVID-19 pulmonary histopathology. A: thick hyaline membranes (arrow), H&E stain, original magnification 400x; B: hemorrhagic areas (arrows), H&E stain, original magnification 20x; C: type II pneumocyte hyperplasia and syncytial cells (white arrows), and casts of desquamated respiratory epithelium (black arrow) in the alveoli, H&E stain, original magnification 400x; D: viral cytopathic effect with multinucleated cells (white arrow) and giant mononucleated cells (black arrows), H&E stain, original magnification 400x; E: perivascular multinucleated cells (arrow), H&E stain, original magnification 400x; F: endotheliitis - reactive endothelial cells (white arrows) and areas of endothelial desquamation (black arrow), H&E stain, original magnification 400x; G: focal fibroblast proliferation and alveolar space obliteration - organizing pneumonia (arrow), H&E stain, original magnification 100x; H: degenerative and necrotic changes in peripheral arterioles (arrows), H&E stain, original magnification 400. H&E: hematoxylin and eosin.

Histologically the lungs revealed evidence of acute respiratory distress syndrome (alveolar hyaline membranes), interstitial (viral) pneumonia, and diffuse zones of hemorrhages (Figures 1A, 1B). There was hyperplasia of type II pneumocytes, with the formation of cytopathic syncytial cells and severe desquamation of bronchial respiratory epithelium as well as two different sets of multinucleated cells (Figures 1C-1E).

The most prevalent multinucleated cells fit the already described morphology of COVID-19-associated changes and were all located within the alveolar spaces (Figure 1D). These cells had a relatively ground-glass opacity cytoplasm with peripheral inclusions, oval to slightly elongated shape, and up to six nuclei and central ruby-red nucleoli, but were however predominantly binucleated. The cellular size was measured to vary between 20 and 40 micrometers.

The second group of multinucleated cells noted was located predominantly in the connective tissue surrounding medium-sized blood vessels (Figure 1E). These cells were significantly smaller, ranging up to 20 micrometers, with irregular scant basophilic cytoplasm, elongated nuclei, ranging from three to seven, with central ruby-red nucleoli.

**Figure 2:**
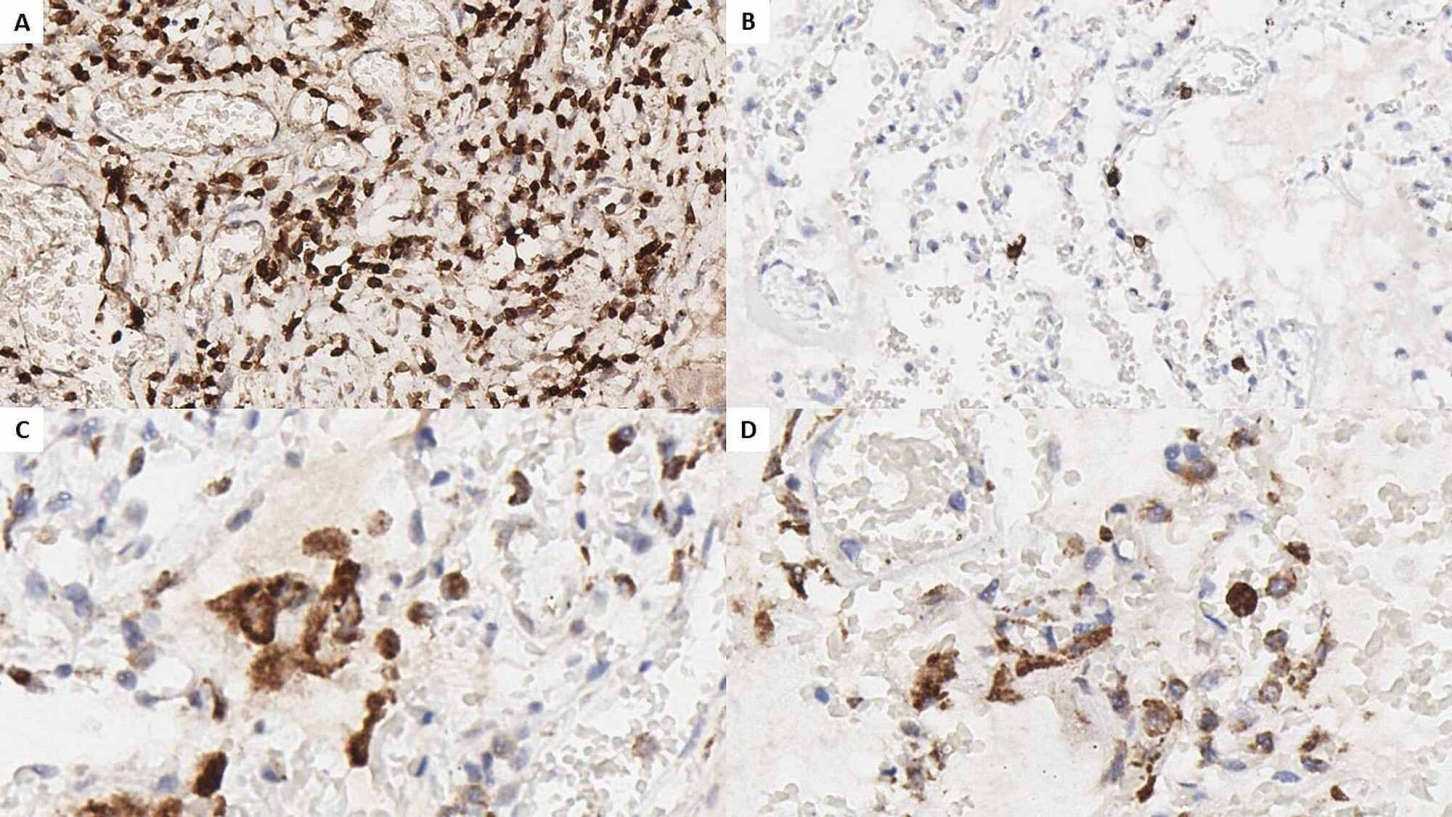
Pulmonary IHC. A: CD3 marking for T lymphocytes, original magnification 200x; B: CD20 marking for B lymphocytes, original magnification 200x; C: CD68 marking for macrophages, reaction in mono and multinucleated cells in the alveolar spaces, original magnification 400x; D: CD68 marking for macrophages, reaction in mono and multinucleated cells in the perivascular connective tissue, original magnification 400x. IHC: immunohistochemistry; CD: cluster of differentiation.

Immunohistochemistry (IHC) was performed with cluster of differentiation (CD) markers to establish the type of lymphocytes present in the lung interstitium, as well as the origin of the two types of giant cells. CD3 was performed for T lymphocytes, CD20 for B lymphocytes, and CD68 for macrophages (Figure 2). The majority of interstitial lymphocytes were T lymphocytes, with a T to B lymphocyte ratio over 10:1 (Figures 2A, 2B). Both fractions of multinucleated cells expressed CD68, confirming their macrophage origin.

Other changes also noted within the pulmonary parenchyma consistent with the already described phenomena in the literature included microthrombi, diffuse endotheliitis, and endothelial desquamation, and areas of fibroblastic proliferation - organizing pneumonia (Figures 1F, 1G). In peripheral areas of the lung, diffuse areas with degenerative and necrotic changes (akin to hyalinization) of the arterial blood vessels were observed (Figure 1H).

Olfactory bulb histopathology

On gross evaluation, the olfactory bulbs appeared to be edematous, with a more pronounced oval shape. On histological examination, severe changes were observed.

**Figure 3:**
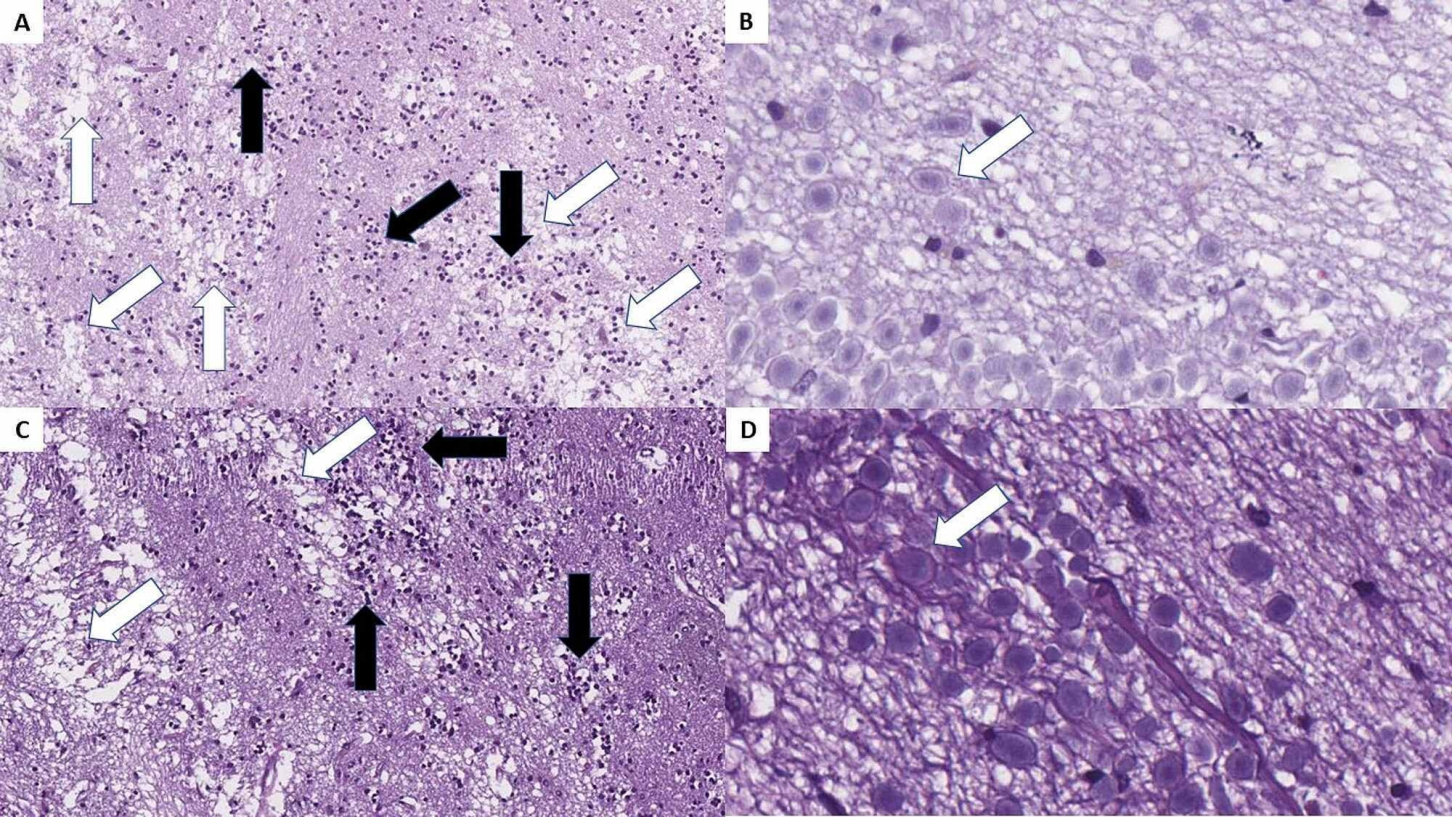
Necrotizing olfactory bulbitis as observed in both cases. A and C: severe edema (white arrows) and diffuse inflammatory cell infiltration (black arrows), H&E stain, original magnification 100x; B and D: diffuse degenerative changes, H&E stain, original magnification 400x. H&E: hematoxylin and eosin.

The normal histological structure of the olfactory bulb was wiped out. There were diffuse edematous areas, inflammatory cell infiltration, and severe neuronal degeneration, and neuronal necrosis - necrotizing olfactory bulbitis. Microglial nodules and diffusely spread degenerative neurons were also noted in the ganglion cell regions (Figure 3).

Central nervous system histopathology

**Figure 4:**
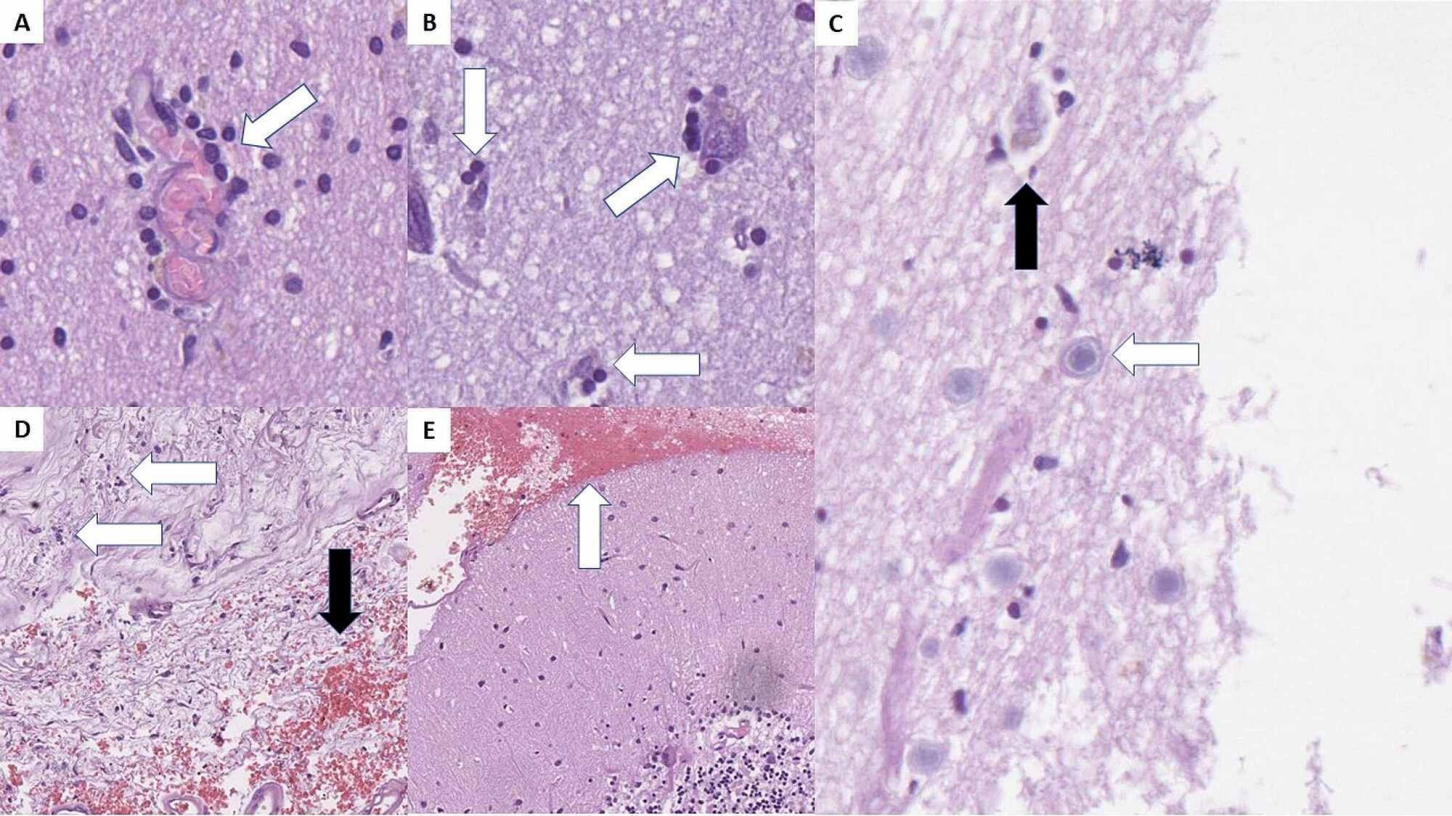
Central nervous system histopathology. A: inflammatory cell cuffs around small blood vessels (arrow), H&E stain, original magnification 400x; B: neuronophagia - inflammatory cells surrounding degenerative neurons (arrows), H&E stain, original magnification 400x; C: Cowrdy type A body (white arrow) and degenerative neuron with neuronophagia (black arrow), H&E stain, original magnification 400x; D: serous meningitis with focal inflammatory cell infiltration (white arrows) and focal hemorrhages (black arrow), H&E stain, original magnification 100x; E: subarachnoid hemorrhage (arrow) in the posterior fossa, H&E stain, original magnification 100x. H&E: hematoxylin and eosin.

Gross changes in the central nervous system included cyanotic and edematous meninges. Histopathology showed encephalitis in the frontal lobes, with lymphocytic cuffs surrounding the small blood vessels and small petechial hemorrhages as well as mild neuronophagia and neuronal degeneration (Figures 4A, 4B).

No inflammatory changes were noted in the remaining brain parenchyma. A small subarachnoid hemorrhage was observed in the posterior fossa of the first case and the frontal area of the second one, as well as mild serous meningitis with focal hemorrhages (Figures 4C and 4D).

Renal histopathology

**Figure 5:**
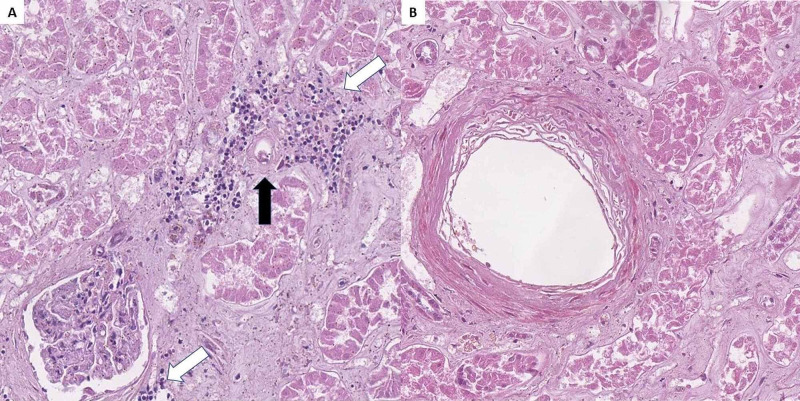
Renal histopathology. A: lymphoplasmacytic inflammatory infiltration (white arrows) surrounding a small blood vessel with fibrinoid necrosis (black arrow), H&E stain, original magnification 200x; B: medium-sized blood vessel with fibrinoid necrosis, H&E stain, original magnification 200x. H&E: hematoxylin and eosin.

Kidney changes were observed only in the first case, with interstitial infiltration by lymphocytes, tubular epithelial cell necrosis, fibrinoid necrosis of blood vessels, and microthrombi in small vessels (Figure 5). All findings present in acute kidney injury. There were also erythrocyte casts in some of the tubules - erythrocyturia, as well as ballooned glomeruli with mild mesangial expansion akin to diabetic nephropathy class IIa.

## Discussion

Both patients had classical symptoms of COVID-19 and a large portion of the gross and histological changes described in the literature thus far [3-5]. The histopathology of the pulmonary parenchyma is highly representative of the already published COVID-associated changes [1,3-5,9-11].

Worthy of notice is the observed cytopathic effect in the type II pneumocytes and the presence of both high levels of T lymphocytes, likely cytotoxic ones, as well as macrophages. These changes further underline the severity of intracellular changes caused by the SARS-CoV-2 viral infection and the degree of the immune response to these changes [12].

Some of the reported features such as the necrotic and degenerative changes seen in the vasculature of the lung in the presented case further support the need for further and prolonged medical care for COVID-19 patients with interstitial pneumonia.

Of significant interest from the cases are the changes in the olfactory bulb. In the presented case we found inflammation, edema, degeneration, and neuronal necrosis. This picture of necrotizing olfactory bulbitis has not been described previously in association with other disorders in humans. Similar histopathological correlations as the ones noted in the olfactory bulbs have been depicted only in primates infected with equine herpesvirus 9, with Cowdry type A cytopathic effect in neurons, which bear some resemblance to the observed neuronal degeneration in our cases and may present another type of cytopathic effect of SARS-CoV-2 [13].

The inflammatory cell infiltration, scattered edema, and neuronal necrosis can be interpreted as a result of acute disease. On the other hand, the degenerative neurons are related to long-lasting damage, from a week to a month. From a clinical standpoint, we cannot properly state the duration of the infection in the patients. Based on histology, however, due to the fibroblastic proliferation in the lungs, we can be quite confident that the duration of the infection was more than a week.

It is generally accepted that virus-induced anosmia is due to nasal congestion and airflow restriction or direct damage to the receptor cells, despite some research pointing to the olfactory bulb as key in disease dissemination [6-8,14-17]. For other coronaviruses, it has been established that they show direct receptor cell tropism [15]. Specifically, for COVID-19-related olfactory dysfunction injury to the sustentacular and other support cells was also envisaged as a probable pathophysiological mechanism, rather than to the olfactory sensory neurons itself [15]. The observed necrotizing olfactory bulbitis in the presented cases may present a new probable morphologic correlate, explaining the development of anosmia in COVID-19 patients.

If the mechanisms of dissemination of the infection by neighboring structures are confirmed, the sustentacular cells would first affect the meninges and the olfactory bulb and the brain after that [15]. This can in part be supported by our finding of diffuse serous meningitis, necrotizing olfactory bulbitis, and encephalitis only in the frontal lobe [13-15].

To our knowledge, this is the first report of histological changes in the olfactory bulbs. So far involvement of the bulb has only been confirmed on imaging modalities, showing either edema or atrophy [18,19]. The development of severe inflammation and neuronal degeneration and necrosis as a result of the viral infection may explain why anosmia is with a sudden onset and takes longer than the other symptoms to resolve [15,16,19]. There is already growing evidence in the field of adult olfactory bulb neurogenesis for the production of new neurons, which replace and integrate into existing chains [20].

## Conclusions

The efforts of the medical and scientific community worldwide during the last year have led to rapid growth in the knowledge on histopathology of COVID-19 infection. So far mainly the pulmonary changes have been reported in detail. Olfactory bulb changes have also been speculated, but have been understudied so far. The described necrotizing olfactory bulbitis depicts another potential mechanism of olfactory alterations in COVID-19 patients.
